# Scalar on time-by-distribution regression and its application for modelling associations between daily-living physical activity and cognitive functions in Alzheimer’s Disease

**DOI:** 10.1038/s41598-022-15528-5

**Published:** 2022-07-07

**Authors:** Rahul Ghosal, Vijay R. Varma, Dmitri Volfson, Jacek Urbanek, Jeffrey M. Hausdorff, Amber Watts, Vadim Zipunnikov

**Affiliations:** 1 Department of Biostatistics, Johns Hopkins Bloomberg School of Public Health, Baltimore, MD USA; 2 National Institute on Aging (NIA), National Institutes of Health (NIH), Baltimore, MD USA; 3 Neuroscience Analytics, Computational Biology, Takeda, Cambridge, MA USA; 4 Department of Medicine, Johns Hopkins University School of Medicine, Baltimore, MD USA; 5 Center for the Study of Movement, Cognition and Mobility, Neurological Institute, Tel Aviv Sourasky Medical Center, Tel Aviv, Israel; 6 Department of Psychology, University of Kansas, Lawrence, KS USA; 7 Department of Physical Therapy, Sackler Faculty of Medicine, and Sagol School of Neuroscience, Tel Aviv University, Tel Aviv, Israel; 8 Rush Alzheimer’s Disease Center and Department of Orthopedic Surgery, Rush University Medical Center, Chicago, IL USA

**Keywords:** Statistical methods, Statistics, Translational research

## Abstract

Wearable data is a rich source of information that can provide a deeper understanding of links between human behaviors and human health. Existing modelling approaches use wearable data summarized at subject level via scalar summaries in regression, temporal (time-of-day) curves in functional data analysis (FDA), and distributions in distributional data analysis (DDA). We propose to capture temporally local distributional information in wearable data using subject-specific time-by-distribution (TD) data objects. Specifically, we develop scalar on time-by-distribution regression (SOTDR) to model associations between scalar response of interest such as health outcomes or disease status and TD predictors. Additionally, we show that TD data objects can be parsimoniously represented via a collection of time-varying L-moments that capture distributional changes over the time-of-day. The proposed method is applied to the accelerometry study of mild Alzheimer’s disease (AD). We found that mild AD is significantly associated with reduced upper quantile levels of physical activity, particularly during morning hours. In-sample cross validation demonstrated that TD predictors attain much stronger associations with clinical cognitive scales of attention, verbal memory, and executive function when compared to predictors summarized via scalar total activity counts, temporal functional curves, and quantile functions. Taken together, the present results suggest that SOTDR analysis provides novel insights into cognitive function and AD.

## Introduction

Wearables are electronic sensors which can be worn as accessories and provide almost real-time continuous streams of user-specific physiological data such as minute-level step counts, heart rate (beats per minute via PPG) and heart rhythm (via ECG), brainwave (EEG), and many others. This rich source of information can be analyzed for a deeper understanding of human behaviours and their influence on human health and disease. For example, wearable physical activity (PA) monitors provide continuous and objective measurements of PA of individuals in their free-living environment^1,2^. The diverse applications of wearable data in biosciences include studies of aging^3,4^, circadian rhythms^5^, estimation of gait parameters and their application in clinical trials^6,7^, comparing patterns and intensity of physical activity between different clinical groups^8,9^ among many others.

In many epidemiological and clinical studies, wearable data is summarized via scalar summaries such as total log activity count (TLAC)^3^, minutes of moderate-to-vigorous-intensity physical activity (MVPA)^3,10^, active-to-sedentary transition probability (ASTP)^11,12^ and others. Scalar summaries, although useful for a particular problem of interest, can often ignore temporal and/or distributional information in continuous streams of data. Temporal or time-of-day information in wearable data can be accounted for using functional data analysis (FDA) approaches that treat wearable data streams as functional observations recorded over 24 hours^5,13–15^. Temporal effects of scalar predictors on physical activity can be captured via function-on-scalar regression and generalized multilevel function-on-scalar regression^16^. Scalar outcomes of interest, e.g., health or disease status can be modelled via scalar-on-function regression models^17,18^ using diurnal physical activity curves as functional predictors typically averaged across the days of observation.

Distributional information in wearable data can be accounted for using distributional data analysis (DDA). Distributions can be encoded via subject-specific histograms^19^, subject-specific quantile functions^20–22^ or subject-specific densities^23–27^. The quantile-function based representation of information in wearable data allows us to model not just mean, but all other quantile-based distributional aspects of wearable data such as variability, skewness, and others. Ghosal et al.^21^ developed a scalar-on-quantile function regression framework (SOQFR) for modelling scalar outcomes of interest based on subject specific quantile functions of wearable data. Matabuena and Petersen^22^ used quantile-function representation for NHANES (2003-2006) accelerometer data to predict health outcomes using survey weighted nonparametric regression models. Talská et al.^28^ developed a compositional scalar-on-function regression method using a centred log ratio transformation^29^ of subject-specific densities. In this article, we propose to use time-by-distribution data objects that capture temporally local distributional information in the user-specific wearable data. In previous work, Horváth et al.^30^ proposed a statistical testing framework for detecting a change in a sequence of distributions, but the distributions were coming from the same unit (monthly financial returns of the same stock). Sharma and Greig^31^ considered distributions over space by time domain and modelled the change over time as linear with respect to the Wasserstein distance. Our approach is different in modelling subject-specific time-by-distribution objects that may have non-linear effects on the outcome with respect to time. Note that two different subjects could have markedly different diurnal patterns of activity but similar distributions. The proposed time-by-distribution data object captures both temporal and distributional aspects of subject-specific PA patterns. Treated as bivariate functional summaries of PA, TD objects can be further used in penalized scalar-on-function regression (SOFR)^32^ for modelling scalar outcomes of interest. We use a penalized bivariate SOFR approach, which simultaneously identifies time of the day and quantile levels of subject-specific PA distribution associated with outcomes of interest. In addition, we employ decompositions of quantile functions via Legendere polynomials and corresponding L-moments^33^ that connect quantile and moment based representations of distributions. This connection enables a decomposition of TD objects via novel diurnal time-varying L-moments.

We are motivated by the application of wearable data in the study of Alzheimer’s Disease (AD) and cognitive performance among older adults. AD is one of the most rapidly growing neurodegenerative diseases in the world. The high prevalence of AD and AD-related death in developed countries can be partially attributed to low levels of physical activity (PA) and sedentary lifestyles^34^. In the absence of any currently existing cure for AD, there is growing interest in identifying cost-effective biomarkers for early identification of risk for AD. Non-invasive, cost-efficient biomarkers are essential for improving early diagnosis of AD^35^. “Digital” biomarkers from sensor and mobile/wearable devices^36^ offer an alternative to existing fluid and imaging markers and there is a growing body of evidence which suggests PA changes might precede clinical manifestation of the disease itself. Physical activities, including activities of everyday living (ADLs), are dependent on mobility and cognitive functioning. Several prospective longitudinal studies have identified physical inactivity as a risk factor for dementia^37–40^. Older adults generally spend most of their waking time in sedentary activities^41^ and individuals with Alzheimer’s disease (AD) have been found to be even less active in previous studies^42^.

In our motivating study by Varma and Watts^8^, physical activity was monitored continuously for seven days using body-worn accelerometers in older adults with mild AD and cognitively normal controls (CNC). Mild AD was found to be associated with reduced moderate-intensity physical activity, reduced peak activity but not with increased sedentary activity or reduced low-intensity physical activity. Although prior research has focused on exploring effects of mild AD on diurnal patterns of PA^8^ and on average or IIV (intra-individual variability) of PA across days^9^, we are interested in whether temporally local distributional information in PA profiles can be used to differentiate between CNC and mild AD and explain cognitive performance.

The article is organized as follows. In “Motivating study” section, we present the background of our motivating study. In  “Modelling framework” section, we present our modelling framework and illustrate some existing approaches for modelling scalar outcomes via scalar, temporal and distributional summaries of wearable PA data. In “Scalar on time-by-distribution regression” section, we introduce time-by-distribution PA data objects and describe the proposed estimation approach using penalized bivariate scalar-on-function regression. In addition, an alternative representation of TD objects via diurnal time-varying L-moments is introduced. In “Application of SOTDR to modelling cognitive status and function in Alzheimer’s disease” section, we demonstrate applications of the proposed method in an Alzheimer’s disease (AD) study and provide comparisons with existing approaches. “Discussion” section concludes with a discussion of the findings, limitations and some possible extensions of the approach.

## Motivating study

### Study participants

Mild AD and cognitively normal control (CNC) participants were recruited by the University of Kansas Alzheimer’s Disease Center Registry (KU-ADC). The study protocol was approved by the KU Medical Center Institutional Review Board. All methods were performed following the relevant guidelines and regulations. Informed consent was obtained from all subjects. A detailed description of recruitment and evaluation of participants in the KU-ADC have been previously reported in Graves et al.^43^ All participants received annual cognitive and clinical examinations, and experienced clinicians trained in dementia assessment provided consensus diagnoses (see “Cognitive status and psychometric test battery” section below for more details). The study sample consisted of individuals with mild AD, defined as a clinical dementia rating (CDR;^44^) scale scores of 0.5 (very mild) or 1 (mild), and control participants, defined as a CDR score of 0. A total of 100 community-dwelling older adults with and without mild AD were recruited. Out of them, N=92 had valid actigraphy data (n = 39 mild AD; n = 53 controls). Descriptive summaries of participant demographics are displayed in Table 1. Age, sex, and years of formal education were reported by either the participant or study partner. The details about other measures are provided in Graves et al.^43^.

**Table 1:** Summary statistics for the complete, AD and CNC samples.

Characteristic	Complete sample		AD		CNC		*P* value
	Mean/Freq	SD	Mean/Freq	SD	Mean/Freq	SD	
Age	73.36	7.11	73.59	7.92	73.19	6.53	0.797
% Female	52.17	N/A	28.20	N/A	69.81	N/A	< 0.001
Years of edu	16.56	3.24	15.53	2.77	17.32	3.38	0.0064
BMI	26.78	4.52	27.28	5.04	26.42	4.11	0.3892
VO2 max	21.99	5.34	21.61	5.24	22.24	5.43	0.592

No statistical difference between the AD and CNC groups are observed across age, BMI, or V0_2_ max. However, AD group had a smaller percentage of females (28.2 vs 69.8 for CNC) and lower education (15.5 years vs 17.3 years for CNC).

### Physical activity

Activity counts were produced by a GT3x+ tri-axial accelerometer. A detailed description of accelerometry measurement can be found in^8^. Briefly, the GT3x+ (Pensacola FL; Actigraph, 2012; 30 Hz sampling rate) is a triaxial accelerometer validated across a range of community dwelling older adults. The accelerometer was placed on the dominant hip of the participants via an elastic belt and the participants were instructed to wear the device 24 hours a day for seven days. Activity counts, collected every second from medio-lateral (ML; front-to-back), antero-posterior (AP; side-to-side), and vertical (VT; rotational) axes were quantified into a single tri-axial composite metric known as vector magnitude^45^, calculated as $VM=\sqrt{ML^2+AP^2+VT^2}$. Average vector magnitude was then computed by aggregating VM (averaging) for each second into minute level activity.

### Cognitive status and psychometric test battery

Cognitive status of the participants were determined through consensus diagnosis by trained clinicians using comprehensive clinical research evaluations and a review of medical records following NINCS-ADRDA criteria^46^. Cognitive tests were administered by a trained psychometrician. The cognitive test battery included tests of verbal memory (Wechsler Memory Scale (WMS)–Revised Logical Memory I and II, Free and Cued Selective Reminding Task), attention (Digits Forward and Backward, Wechsler Adult Intelligence Scale (WAIS) subscale Letter– Number Sequencing) and executive function (Digit Symbol Substitution Test, and Stroop Color–Word Test (interference score), Trail Making Test Part B, and Category Fluency). Composite scores for each domain (verbal memory (VM), attention (ATTN), and executive function (EF)) were derived using confirmatory factor analysis (CFA), a flexible approach for summarizing multiple cognitive scores into empirically and theoretically justified components. Scores were standardized to the mean performance of CNC participants. Additional information on the CFA derived factor scores can be found in Varma et al.^7^.

## Modelling frameworks

Suppose, we have minute-level wearable observations such as activity counts or the number of steps per minute denoted by $$X_{ij}(t) $$ for subject $$i=1,\ldots ,n$$, on *j*-th day, $$j=1,\ldots ,n_i$$, at time *t*, $$t=1,2,\ldots ,1440$$. We denote by $$Y_i$$ a scalar outcome of interest such as a cognitive status or a score on a psychometric test that can be continuous or discrete and we assume it comes from an exponential family. We also denote by $$\mathbf {Z}_i$$ a vector of covariates. In this section, we review three existing modelling approaches that relates $$Y_i$$ and $$X_{ij}(t)$$ including a simple Generalized Linear Model (GLM) regression using scalar summaries of wearable observations, functional data regression of temporal (time-of-day) curves, and distributional data regression using subject-specific quantile functions.

### GLM regression using subject-specific scalar summaries

**Figure 1:**
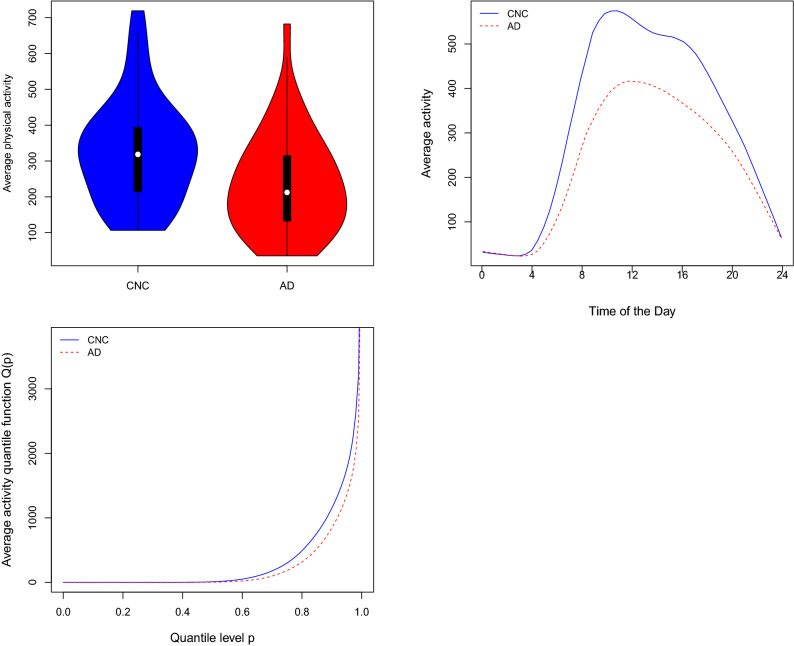
Top left: violin plot of subject-specific averages for CNC and AD participants. top right: smoothed diurnal activity profiles averaged across CNC (blue) and AD (red) participants. Bottom left: average quantile functions of physical activity for AD and CNC participants.

In this approach, the scalar response variable $Y_i$ is modelled via a subject-specific scalar summary of wearable observations aggregated across all times and days. Examples include a total mean as a measure of tendency, a standard deviation as a measure of variability, minutes spent in activities of certain intensity such as light or moderate-to-vigorous, and others. For example, subject-specific average activity count ${\bar{X}}_i=\frac{1}{1440n_i} \sum _{j=1}^{n_i}\sum _{t=1}^{1440}X_{ij}(t)$. The top left panel of Fig. 1 displays the distribution of subject-specific averages for CNC (blue) and AD (red) groups in our study.

We observe that participants with AD on average, have a lower mean physical activity level compared to CNC. There is also significant overlap between the two distributions and they are not clearly separable using this PA metric. To formally model this, a generalized linear model (GLM) can be used

1 $$\begin{aligned} E(Y_i|\mathbf {Z}_i,{\bar{X}}_i)=\mu _i, \quad g(\mu _i)=\alpha +\mathbf {Z}_i^T\varvec{\gamma } + {\bar{X}}_i\beta , \end{aligned}$$

where a scalar regression coefficient $\beta$ represents the effect of average PA on the mean of the response of interest $Y_i$ adjusted for covariates $\mathbf {Z}_i$ and $g(\cdot )$ is a known link function (e.g., *logit* or identity).

### Functional data analysis of subject-specific temporal curves

Functional data analysis (FDA) allows us to model temporal aspects in wearable observations $X_{ij}(t)$. To derive subject-specific diurnal minute-level curves, one may average wearable observations across all days at each time-point $t=1,2,\ldots ,1440$ as $X_i(t)=\frac{1}{n_i} \sum _{j=1}^{n_i}X_{ij}(t)$. The top right panel of Fig. 1 displays average smoothed diurnal activity profiles for CNC (blue) and AD (red) groups. It can be noticed that the curve for mild-AD group have a unimodal diurnal shape, compared to a bimodal shape for CNC, and the largest difference between the two groups appears to be in the morning and in the afternoon (during the second peak for CNC). Similar observations were also made by Varma and Watts^8^ during their analysis of this data. To formally model the association with functional predictors, scalar-on-function regression (SOFR)^17^ can be used as follows

2 $$\begin{aligned} E(Y_i|\mathbf {Z}_i, X_i(t))=\mu _i, \quad g(\mu _i)=\alpha +\mathbf {Z}_i^T\varvec{\gamma } + \int _{T}X_i(t)\beta (t)dt, \end{aligned}$$

where the functional regression coefficient $\beta (t)$ captures the time-varying effect of the diurnal curve $X_i(t)$ on the response $Y_i$ and $T=(0,24)$ is the daily 24 hour window. Note that, the average subject-specific PA can be estimated back from the diurnal profile $X_i(t)$ as ${\bar{X}}_i=\int _T X_i(t)dt$, therefore for a constant functional regression coefficient $\beta (t)=\beta$, one gets back the generalized linear model (1) for scalar predictors from model (2).

### Distributional data analysis using subject-specific quantile functions

Distributional data analysis can capture and model distributional aspect of wearable observations via subject-specific probability density functions (pdf), cumulative distribution functions (CDF), or quantile functions^21^. If we ignore the temporal information by suppressing the time index *t*, we can denote by $X_{ik}$, $k=1,\ldots ,m_i$, all wearable observations for subject *i*. We assume $X_{ik}$ follow the same subject-specific distribution defined by subject-specific cumulative distribution function $F_i(x)$, where $F_i(x)=P(X_{ik}\le x)$. Then, we can define the subject-specific quantile function $Q_i(p)= \inf \{x: F_i(x)\ge p\}$. The subject-specific quantile function characterizes the distribution of wearable observations for a specific subject. The subject-specific cdf can be estimated via its empirical counterpart ${\hat{F}}_i(x)=\frac{1}{m_i}\sum _{k=1}^{m_i}I(X_{ik}\le x)$ and subject-specific quantile function can be estimated as ${\hat{Q}}_i(p)={\hat{F}}_i^{-1}(p)$. In this paper, we use the following estimator of quantile functions via a linear interpolation of the order statistics^47^:

$$\begin{aligned} {\hat{Q}}(p)=(1-w)X_{([(n+1)p])}+wX_{([(n+1)p]+1)}, \end{aligned}$$

where $X_{(1)}\le X_{(2)}\le \cdots ,X_{(n)}$ are the corresponding order statistics from a sample $(X_1,X_2,\ldots , X_n)$ and *w* is a weight satisfying $(n+1)p=[(n+1)p]+w$. Note that the subject-specific average of wearable observations $X_{ij}(t)$ can be also estimated from the subject-specific quantile function as ${\bar{X}}_i=\int _{0}^{1} {Q}_i(p)dp$.

The bottom left panel of Fig. 1 displays the average quantile functions of physical activity for the CNC and AD groups. A reduced capacity of physical activity can be observed for the AD samples compared to CNC across upper quantile levels such as $p > 0.75$. Following the approach of Ghosal et al.^21^, the subject-specific quantile functions of PA can be used for modelling $Y_i$ using scalar-on-function regression (SOFR) (3) adjusted for $\mathbf {Z}_i$. SOFR model is as follows

3 $$\begin{aligned} E(Y_i|\mathbf {Z}_i, Q_i(p))=\mu _i, \quad g(\mu _i)=\alpha +\mathbf {Z}_i^T\varvec{\gamma } + \int _{0}^{1}Q_i(p)\beta (p)dp, \end{aligned}$$

where the functional regression coefficient $\beta (p)$ captures the distributional effect of the PA quantile function $Q_i(p)$ on the response of interest $Y_i$. In the case $\beta (p)=\beta$, a constant, one again get back the generalized linear model (1) from model (3), since ${\bar{X}}_i=\int _{0}^{1} Q_i(p)dp$.

Ghosal et al.^21^ re-represented SOFR model for quantile function predictors via L-moments^33^. L-moments are defined as the expectation of a linear combination of order statistics. In particular, the *r*-th order L-moment of a random variable *X* is defined as

$$\begin{aligned} L_r=r^{-1}\sum _{k=0}^{r-1}(-1)^k {r-1 \atopwithdelims ()k} E(X_{r-k:r})\quad r=1,2,\ldots , \end{aligned}$$

where $X_{1:n}\le X_{2:n}\le \cdots \le X_{n:n}$ denote the order statistics of a random sample of size *n* drawn from the distribution of *X*. The first order L-moment, $L_1$, equals the traditional mean. The second order L-moment, $L_2 = 1/2E(X_{2:2}-X_{1:2})$, represents a robust measure of scale, and equals exactly a half of Gini-coefficient or mean absolute difference. The third and fourth order L-moments, $L_3 = 1/3E(X_{3:3}-2X_{2:3}+X_{1:3})$ and $L_4 = 1/4E(X_{4:4}-3X_{3:4}+3X_{2:4}-X_{1:4})$, capture higher-order distributional properties and normalized by $L_2$ can be interpreted similarly to traditional higher-order moments such as skewness and kurtosis. The main advantages of L-moments is the existence of all moments, if first moment exist, their uniqueness and robustness. For SOQFR Ghosal et al.^21^ adapted an alternative representation of L-moments as projections of quantile functions on Legendre polynomial basis, given by

$$\begin{aligned} L_r = \int _0^1 Q(p)P_{r-1}(p)dp. \end{aligned}$$

Here $P_r(p)$ is the shifted Legendre polynomial (LP) of degree *r* defined as

$$\begin{aligned} P_r(p) = \sum _{k=0}^r s_{r,k}p^r, \quad s_{r,k} = (-1)^{r-k}{r \atopwithdelims ()k}{r+k \atopwithdelims ()k} = \frac{(-1)^{r-k}(r+k)!}{(k!)^2(r-k)!}. \end{aligned}$$

The shifted Legendre polynomials form an orthogonal basis of $L_2[0,1]$. Using the LP decomposition for subject-specific quantile functions $Q_i(p) \approx \sum _{k=1}^K(2k-1)L_{ik}P_{k-1}(p)$ and $\beta (p)=\sum _{k=1}^K\beta _kP_{k-1}(p)$, SOFR model can be reduced to a GLM as $g(\mu _i)=\alpha +\mathbf {Z}_i^T\varvec{\gamma } + \int _{0}^{1}Q_i(p)\beta (p)dp = \alpha +\mathbf {Z}_i^T\varvec{\gamma } + \sum _{k=1}^K \beta _kL_{ik}$. This representation of SOFR via L-moments provides both the functional interpretation of significance of $Q_i(p)$ via $\beta (p)$ and the distributional interpretation in terms of the significance of specific L-moments via $\beta _k$.

## Scalar on time-by-distribution regression

In this section, we propose to capture temporally local distributional information in wearable observations using subject-specific time-by-distribution data objects and use bivariate scalar-on-function regression to relate these to a scalar response of interest. We refer to this as scalar on time-by-distribution regression (SOTDR) and also show how two-way TD data objects can be parsimoniously represented via a collection of time-varying L-moments that capture distributional changes over the time-of-day.

### SOTDR via time-by-distribution data objects

We develop quantile-based time-by-distribution data objects that capture the temporally local distributional aspects of wearable observations. The quantile-based time-by-distribution data object is then defined as

$$\begin{aligned} Q_i(t,p)= \textit{p-th quantile of }\{X_{ij}(s)\}_{j=1}^{n_i}, s \in (t-h,t+h). \end{aligned}$$

Here 2*h* is the window length around time *t*. Note that $Q_i(t,p)$ is a bivariate functional summary of subject-specific observational data. For each fixed *t* (time of the day), it provides distributional encoding as a function of quantile-level *p*, e.g., $Q_i(t,\cdot )$ is a quantile function for each *t*. For each fixed *p*, $Q_i(\cdot ,p)$ captures the diurnal pattern of the *p*-th quantile level of wearable observations as a function of time *t*. Note that the subject-specific average PA can be again be estimated back aggregating the bivariate time-by-distribution data objects as ${\bar{X}}_i=\int _{T}\int _{0}^{1} {Q}_i(t,p)dpdt$. For the analysis presented in this paper, we fix total window length $2h=10$ minutes (i.e., $h=5$), but any other window lengths can be used as well. Since the sample considered in this study is highly sedentary^9^, a window length of 10 minutes still retains the diurnal patterns of PA without any significant loss of information.

**Figure 2:**
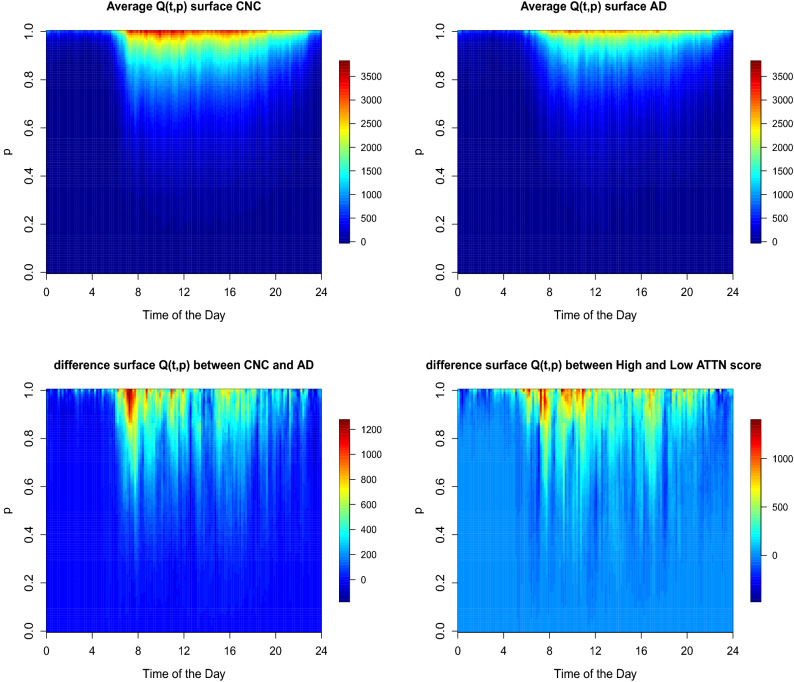
The average bivariate time-by-distribution PA surface $Q_i(t,p)$ for CNC (top left) and AD (top right) groups. The difference between CNC and AD (bottom left) and the difference between subjects with high (above $75\%$ percentile) and low (below $25\%$-percentile) of cognitive attention (ATTN) scores (bottom right).

Figure 2 displays the heatmaps of average time-by-distribution surfaces $Q_i(t,p)$ for CNC (top left) and AD (top right), the difference between them (bottom left). One can see that the largest differences between the two groups exist during the morning (8 a.m.–11 a.m.) and in afternoon (3 p.m.–5 p.m.) across the upper quantile levels ($p>0.6$). At the bottom right panel of Fig. 2 we plot the heatmap of difference in time-by-distribution surfaces $Q_i(t,p)$ between the participants with high (above $75\%$-percentile) and low (below $25\%$-percentile) cognitive scores of attention (ATTN) in a combined sample including subjects from both AD and CNC groups. Overall, TD encoding of physical activity is clearly more informative than just temporal or just distributional information from Fig. 1.

To formally model the association of TD data objects with a scalar response, we propose to use them as predictors in two-way scalar-on-function regression (SOFR) as follows:

4 $$\begin{aligned} E(Y_i|\mathbf {Z}_i, Q_i(t,p))=\mu _i, \quad g(\mu _i)=\alpha +\mathbf {Z}_i^T\varvec{\gamma } + \int _{0}^{1} \int _{T}Q_i(t,p)\beta (t,p)dtdp. \end{aligned}$$

Here $\beta (t,p)$ represents the bivariate functional regression coefficient that captures both the temporal and distributional effect of $Q_i(t,p)$ on the response of interest $Y_i$. As before, with the constant regression $\beta (t,p)=\beta$, the bivariate SOFR model (4) reduces to the generalized linear model (1) for scalar predictors. The estimation approach of this model is discussed below.

### Estimation of the time-by-distribution regression coefficient $\beta (t,p)$

We follow a two-step estimation approach for the bivariate SOFR model (4) in the paper. In step 1, we model the bivariate regression functional coefficient $\beta (t,p)$ using a tensor product of univariate cubic B-spline basis functions of both temporal and quantile level arguments, *t* and *p*. Suppose, $\{B_{T,k}(t)\}^{K_0}_{k=1}$ and $\{B_{P,\ell }(p)\}^{L_0}_{\ell =1}$ are the set of known basis functions over *t* and *p*, respectively. Then, $\beta (t,p)$ is modelled as $\beta (t,p)=\sum _{k=1}^{K_0}\sum _{\ell =1}^{L_0}\theta _{k,\ell }B_{T,k}(t)B_{P,\ell }(p)$. Using this expansion model (4) is reformulated as

5 $$\begin{aligned} g(\mu _i)= & {} \alpha +\mathbf {Z}_i^T\varvec{\gamma } + \sum _{k=1}^{K_0}\sum _{\ell =1}^{L_0}\theta _{k,\ell }\int _{T} \int _{0}^{1}Q_i(t,p)B_{T,k}(t)B_{P,\ell }(p)dtdp\nonumber \\= & {} \alpha +\mathbf {Z}_i^T\varvec{\gamma } +\mathbf {W}_{i}^T\varvec{\theta }, \end{aligned}$$

where we denote by $\mathbf {W}_i$ the $K_0L_0$-dimensional stacked vectors of $\{\int _{0}^{1}Q_i(t,p)B_{T,k}(t)B_{P,\ell }(p)dtdp\}_{k=1,\ell =1}^{K_0,L_0}$ and $\varvec{\theta }$ is the corresponding $K_0L_0$-dimensional vector of unknown basis coefficients $\theta _{k,\ell }$’s. Thus, the model (5) can be seen as a GLM with subject specific predictors $\mathbf {W}_{i}^{k,\ell }=\int _{0}^{1}Q_i(t,p)B_{T,k}(t)B_{P,\ell }(p)dtdp$. We use a penalized negative log-likelihood criterion with LASSO^48^ penalty on the coefficients, which selects only those $\mathbf {W}_{i}^{k,\ell }$ which influences the response of interest $Y_i$. This effectively helps to reduce the number of parameters in the model (especially important because of a relatively small sample size $n = 92$) and allows a sparse representation of the functional regression coefficient $\beta (t,p)$. The penalized negative log likelihood criterion is given by

6 $$\begin{aligned} S(\psi )=R(\alpha ,\varvec{\gamma },\varvec{\theta })= -2log L(\alpha ,\varvec{\gamma },\varvec{\theta };Y_i,\mathbf {Z}_i,\mathbf {W}_i) + \lambda ||\varvec{\theta }||_{1}. \end{aligned}$$

In step 2, the selected predictors $\mathbf {W}_{i}^{k,\ell }$ (with non-zero coefficients) are used in the GLM (5) without any penalization (this also overcomes penalization bias of LASSO) for inference. The estimated regression coefficient function is then given by ${\hat{\beta }}(t,p)=\sum _{k=1}^{K_0}\sum _{\ell =1}^{L_0}{\hat{\theta }}_{k,\ell }B_{T,k}(t)B_{P,\ell }(p)$ (note that ${\hat{\theta }}_{k,\ell }=0$ if $\mathbf {W}_{i}^{k,\ell }$ is not selected in the first step).

### SOTDR-L: SOTDR via time-varying L-moments

Following Ghosal et al.^21^ who adapted L-moments to SOFR with quantile function predictors, we adapt L-moments to SOTDR by introducing subject-specific time-varying L-moments $L_{ir}(t)$ that depend on the time of the day *t*. Specifically, we define the diurnal time-varying *r*-th order L-moment for subject *i* as

$$\begin{aligned} L_{ir}(t)= \textit{r-th L-moment of }\{X_{ij}(s)\}_{j=1}^{n_i}, s \in (t-h,t+h). \end{aligned}$$

Here we again consider a window of total length 2*h* centered at time *t*. The diurnal time-varying $L_{ir}(t)$ curves capture the temporal change of the subject-specific distribution. For example, the first order time-varying L-moment $L_{i1}(t)$ simply represents the diurnal mean curve $X_i(t)$ aggregated into 10 minutes epoch (for $h=5$). The second order time-varying L-moment $L_{i2}(t)$ captures a temporal change in variability and is similar to the diurnal standard deviation curve of physical activity considered by^8^.

**Figure 3:**
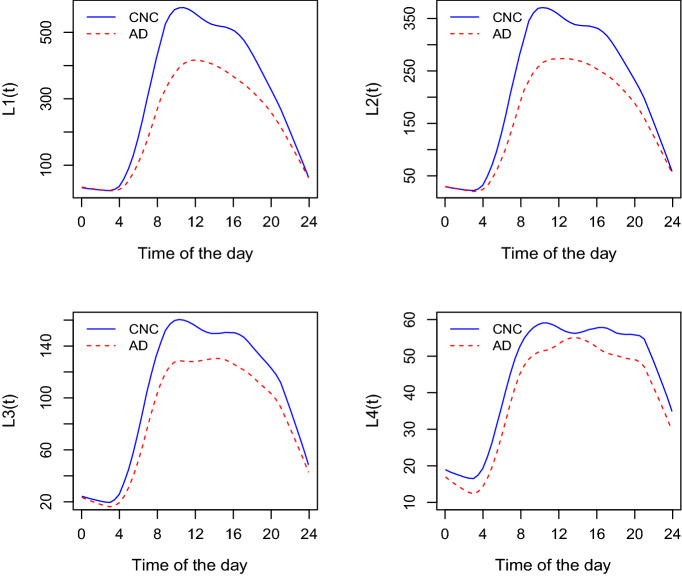
The first four time-varying L-moments of daily physical activity averaged within CNC (blue) and AD (red) groups.

Figure 3 displays the first four time-varying L-moments $L_r(t)$ of physical activity, averaged within CNC (blue) and AD (red) groups. Note that the first time-varying L-moments $L_1(t)$ exactly equal to the temporal diurnal curves from the top right panel of Fig. 1. Subject-specific *r*-th order time-varying L-moment $L_{ir}(t)$ is related to the time-by-distribution PA data object $Q_i(t,p)$ through its projection on Legendre polynomial basis $P_{r-1}(p)$ as follows

$$\begin{aligned} L_{ir}(t) = \int _0^1 Q_i(t,p)P_{r-1}(p)dp. \end{aligned}$$

One can notice that mild AD has lower $L_1(t)$, $L_2(t)$, $L_3(t)$, and $L_4(t)$ moments compared to the CNC, particularly in the morning and somewhat in the afternoon.

We propose to use the time-varying subject-specific L-moments $L_{ir}(t)$ for modelling $Y_i$ using an additive SOFR model. If the shifted Legendre polynomials $P_{\ell -1}(p)$ are used as the basis in *p* for modelling the bivariate functional effect $\beta (t,p)$, the additive SOFR model (7) in terms of time-varying L-moments of PA provides an alternative representation of the bivariate SOFR model (4) that is additionally interpretable from distributional point of view. We will refer to this approach as SOTDR-L. In particular, we have,

7 $$\begin{aligned} g(\mu _i)&=\alpha +\mathbf {Z}_i^T\varvec{\gamma } + \int _{0}^{1} \int _{T}Q_i(t,p)\beta (t,p)dtdp \nonumber \\&=\alpha +\mathbf {Z}_i^T\varvec{\gamma } + \int _{T}\sum _{k=1}^{K_0}\sum _{\ell =1}^{L_0}\theta _{k,\ell } B_{T,k}(t) \int _{0}^{1}Q_i(t,p)P_{\ell -1}(p) dt dp \nonumber \\&= \alpha +\mathbf {Z}_i^T\varvec{\gamma } + \sum _{\ell =1}^{L_0}\int _{T}L_{i\ell }(t)\sum _{k=1}^{K_0}\theta _{k,\ell } B_{T,k}(t) dt \nonumber \\&= \alpha +\mathbf {Z}_i^T\varvec{\gamma } + \sum _{\ell =1}^{L_0}\int _{T}L_{i\ell }(t)\beta _{\ell }^{*}(t) dt. \end{aligned}$$

Here the functional regression coefficient $\beta ^{*}_r(t)$ capture the diurnal time-varying effect of the *r*-th order time-varying L-moment on the response $Y_i$ at time *t*. Thus, we get an additive SOFR with time-varying L-moments. It is important to note that if $L_0 = 1$ we get exactly the SOFR model (2) that uses subject-specific temporal curves as predictors. Thus, SODTR-L model (7) strictly includes model (2).

## Application of SOTDR to modelling cognitive status and function in Alzheimer’s disease

In this section, we apply SOTDR to model cognitive status and function in the Alzheimer’s disease (AD) study and compare it to the three existing approaches including a GLM regression with scalar total activity count summary, SOFR with temporal diurnal curves and SOFR with quantile functions.We use penalized spline regression^49^ to estimate the unknown coefficient functions $\beta (t)$ and $\beta (p)$ in SOFR. For both diurnal and distribution modelling, 12 B-Spline basis functions with a second order difference penalty are used. The refund package^50^ in R^51^ is used for implementation of SOFR. First, we will model cognitive status (CNC vs AD) and the three cognitive scores of attention (ATTN), visual memory (VM), and executive function (EF) using the bivariate time-by-distribution data objects as illustrated in the “SOTDR via time-by-distribution data objects” section. Second, we alternatively use an additive SOFR with time-varying L-moments.

### SOTDR modelling of cognitive status

We model cognitive status (CNC vs AD) using the SODTR model (4) with an additive adjustment for age, sex and years of education. For comparison with existing approaches, we fit models (1), (2) and (3) using as predictors subject-specific average PA, diurnal PA curves, quantile PA functions, respectively. Ten-minute diurnal PA curves have been calculated by aggregating minute-level data into 10 minutes epochs, resulting in subject-specific diurnal PA curves $X_i(t)$ of length 144. As mentioned earlier, since the participants of the study were highly sedentary^9^ such 10-minute aggregation serves as pre-smoothing and retains the key temporal patterns of PA. When we report predictive performance summaries such as the area under the curve (AUC) of the receiver operating characteristic, we perform repeated five-fold cross-validation and report the average cross-validated AUC (cvAUC). In Model (4), cross-validated AUC involves only cross-validation of part 2 of the estimation process, that is the same components of $\mathbf {W}$ selected in Step 1 are used in each iteration of the cross validation. It is important to note that for a large dataset this step will not be necessary as $Q_i(t,p)$ could directly be used as a bivariate functional predictor. The results of the analyses are presented in Table 2.

**Table 2:** The results of modelling cognitive status (CNC vs AD) and physical activity using Model 1–4 with an adjustment for age, sex, and education.

	Dependent variable: cognitive status (CNC vs AD)			
	Model 1	Model 2	Model 3	Model 4
Intercept	7.608**	6.549*	10.588**	12.368***
	(3.567)	(3.615)	(4.139)	(4.591)
Age	−0.051	−0.040	− 0.072*	− 0.089*
	(0.038)	(0.039)	(0.043)	(0.047)
Sex	2.134***	2.111***	2.527***	2.637***
	(0.554)	(0.553)	(0.624)	(0.676)
Education	− 0.224**	− 0.213**	− 0.167*	− 0.174*
	(0.091)	(0.091)	(0.092)	(0.095)
${\bar{X}}_i$	− 0.005***	NA	NA	NA
	(0.002)			
$$X_i(t)$$	NA	$${\hat{\beta }}(t)^{**}$$	NA	NA
$$Q_i(p)$$	NA	NA	$${\hat{\beta }}(p)^{**}$$	NA
$$ Q_i(t,p)$$	NA	NA	NA	$${\hat{\beta }}(t,p)^{***}$$
Observations	92	92	92	92
cvAUC	0.781	0.773	0.792	0.811

The standard deviation of the estimated coefficients for the scalar predictors are indicated in the parenthesis. Predictors: model 1-scalar average PA, model 2–diurnal PA curves, model 3-quantile functions, model 4-SOTDR with time-by-distribution data objects.

*$${p}<0.1$$; **$${p}<0.05$$; ***$${p}<0.01$$.

The *p* values for $\beta (t)$ and $\beta (p)$ in SOFR correspond to the *p* values from global test of these coefficients and are as reported by the pfr function for scalar-on-function regression within the refund package^50^ in R^51^. These are based on a test statistic motivated by an extension of Nychka’s analysis^52^ of the frequentist properties of Bayesian confidence intervals for smooths^53^. The *p* values for $\beta (t,p)$ are based on a likelihood ratio test (LRT) (of inclusion) in the second stage of the estimation process with the selected components of $\mathbf {W}$ coming from the first stage.

**Figure 4:**
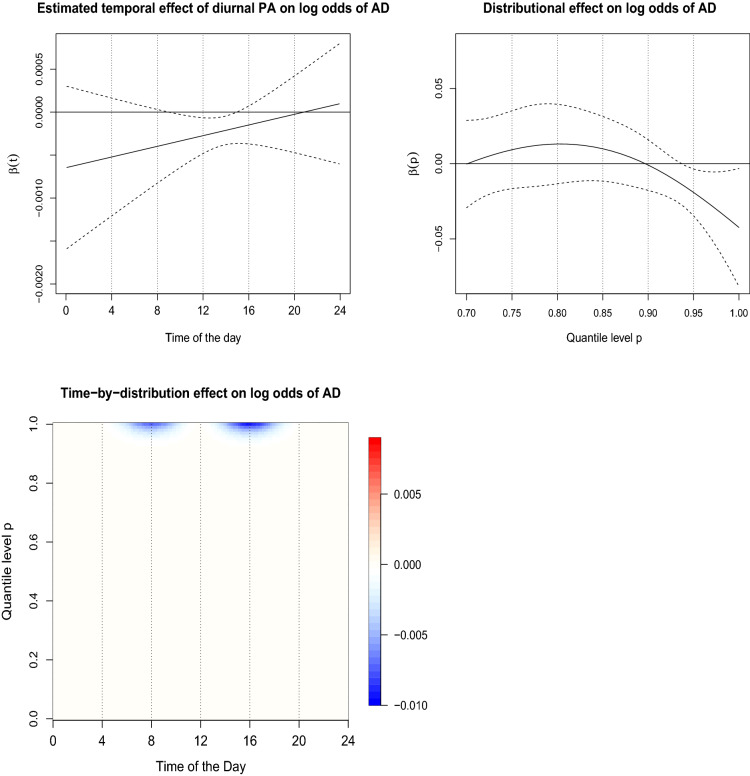
The estimated regression coefficients for Models 2–4. Estimated temporal effect $\beta (t)$ (top left. *t* denoting time of the day). Estimated distributional effect $\beta (p)$ (top right, $p\in (0.7,1)$). Estimated bivariate effect $\beta (t,p)$ of time-by-distribution PA surface (bottom left).

Model (1) shows that higher subject-specific average PA is significantly associated ($\alpha = 0.05$) with a lower odds of AD. The cvAUC value of 0.781 illustrates a satisfactory discriminatory power of the model and is set as a benchmark for comparison with the other three models. The estimated functional regression coefficient $\beta (t)$ for Model (2) illustrating a diurnal effect of PA profile on log-odds of AD is displayed in the top left panel of Fig. 4. Model (2) finds that higher PA during morning hours ($\sim$ 10 a.m.–3 p.m.) is significantly associated ($\alpha =0.05$) with a lower odds of AD^49^. The average cvAUC of 0.773 suggests that, although, the diurnal patterns of average PA offer additional temporal insights, they do not necessarily offer more discrimination between CNC and AD groups compared to the use of simple average PA (Model 1, cvAUC = 0.781). Model (3) finds the significance of subject-specific quantile functions of PA.

The estimated functional regression coefficient $\beta (p)$ for Model (3) illustrating a distributional effect of PA on log-odds of AD is displayed ($\beta (p)$ not significant for $p<0.7$) in the top right panel of Fig. 4 and shows that higher upper quantile levels ($p \in (0.90, 1)$) of PA are significantly associated with lower odds of AD^49^. Increased cvAUC of 0.792 indicates higher discriminatory power of distributional encoding of PA (in particular, maximal PA) between CNC and AD compared to the average PA.

The estimated bivariate functional effect $\beta (t,p)$ for Model (4) is shown in the bottom left panel of Fig. 4. We used $K_0=L_0=12$ cubic B-spline basis functions for modelling $\beta (t,p)$. Increased maximal capacity of PA during the morning ($\sim$ 7 a.m.–10 a.m.) and in the afternoon ($\sim$ 3 p.m.–5 p.m.) is found to be associated with lower odds of AD. An increased cvAUC of 0.811 (around 3.8% gain) illustrates additional discriminatory power of the time-by-distribution PA data objects, while simultaneously capturing temporally local distributional effects of the PA on log-odds of AD.

### SOTDR-L modelling of cognitive status

Next, we illustrate an application of SOTDR-L that uses diurnal time-varying L-moments for modelling cognitive status (CNC vs AD) outcome. For interpretability, we use the first four diurnal L-moments profile $L_{ik}(t)$ ($L_0=4$) as functional predictors and adjust for age, sex and years of education. Since we have a relatively small sample size ($\hbox {n}=92$), we follow a penalized SOFR approach to select the L-moments $L_{ik}(t)$-s, which are most informative.

**Figure 5:**
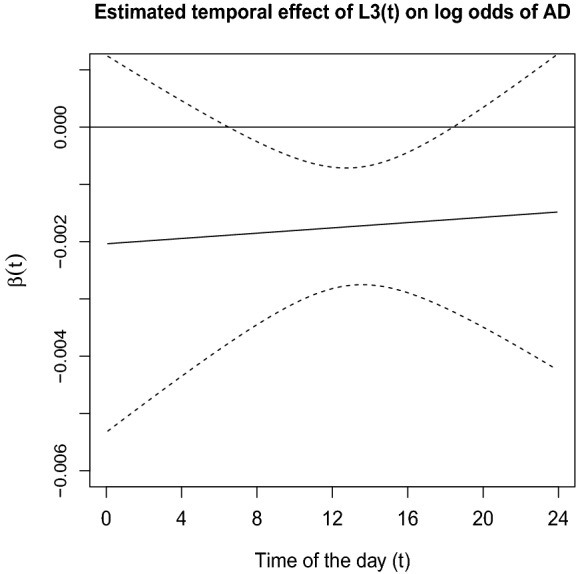
Estimated diurnal effect $\beta (t)$ of $L_{i3}(t)$ of PA on log odds of AD.

In particular, we re-express the SOFR model (7) in terms of functional principal component scores of $L_{ik}(t)$ following a functional principal component regression approach^54,55^,

8 $$\begin{aligned} E(Y_i|\mathbf {Z}_i, \{L_{ir}(t)\}_{r=1}^4)=\mu _i, \quad g(\mu _i)=\alpha +\mathbf {Z}_i^T\varvec{\gamma } + \sum _{r=1}^{4}\sum _{s=1}^{n_r}\xi _{irs}\beta _{r,s}. \end{aligned}$$

Here $\xi _{irs}=\int _{T}L_{ir}(t)\psi _{s}(t)$ is the projection of the diurnal L-moment $L_{ir}(t)$ on the eigenbasis $\psi _{s}(t)$ and $\beta _r(t)$ is modelled as $\beta _r(t)=\sum _{s=1}^{n_r}\beta _{r,s}\psi _{s}(t)$. We use the group exponential Lasso (GEL) penalty^56^ on the basis coefficients $\{\beta _{r,s}\}_{s=1}^{n_r}$ to perform variable selection in order to identify informative time-varying L-moments $L_{ik}(t)$. GEL is a bi-level selection penalty and enjoys the added flexibility of forcing some of the coefficients within a particular group to be zero, thus effectively reducing the number of parameters, which is especially useful in our scenario due to the very low sample size. The proposed variable selection approach selects the 3rd order time-varying L-moments $L_{i3}(t)$ to be most informative i.e, most discriminating between the two groups (CNC and AD) while adjusting for age, sex and years of education. The grpreg package^57^ in R is used for implementing the variable selection method using GEL. The estimated diurnal effect of $L_{i3}(t)$ is shown in Fig. 5. We observe that an increase in the value of third order L-moment of physical activity, during the window (8 a.m.–6 p.m.) is associated with a lower odds of AD. The third order L-moment $L_{i3}(t)$ is related to L-skewness of the PA and its significance is therefore very interesting from a clinical perspective. We also perform repeated cross-validation using $L_{i3}(t)$ as predictor in a SOFR model while adjusting for age, sex, and years of education. An increased cvAUC of 0.802 (around 2.7% gain) illustrates satisfactory discriminatory power of the proposed metric offering both distributional and temporal encoding of physical activity. Likely, because of restricting the number of L-moments and the use of GEL, the temporal findings of SOTDR-L differ from temporal findings of SOTDR. While SOTDR highlights activity in the upper quantile levels during 6–10 a.m. and 2–6 p.m. time periods, SOTDR-L highlights the third order L-moment of activity during mid-day hours that are similar to those from SOFR on temporal diurnal curves. Chosen third order time-varying L-moments in SOTDR-L also seems to result in an increase in cvAUC compared to SOFR that uses temporal diurnal curves (that are equivalent to the first order time-varying L-moments).

### Modelling attention

In this section, we apply SOTDR to model the cognitive score of attention (ATTN) of all the subjects and the results are compared with those from regression with subject-specific average PA, FDA using diurnal PA curves, DDA using quantile functions. Adjusted R-squared, defined as the adjusted proportion of variance explained, where original variance and residual variance are both estimated using unbiased estimators^58^, is used in Models 2-4 for the evaluation of in-sample predictive performance. Cross-Validated R-squared (from repeated 5 fold cross-validation) is reported to compare out-of-sample prediction performance of the different models.

**Figure 6:**
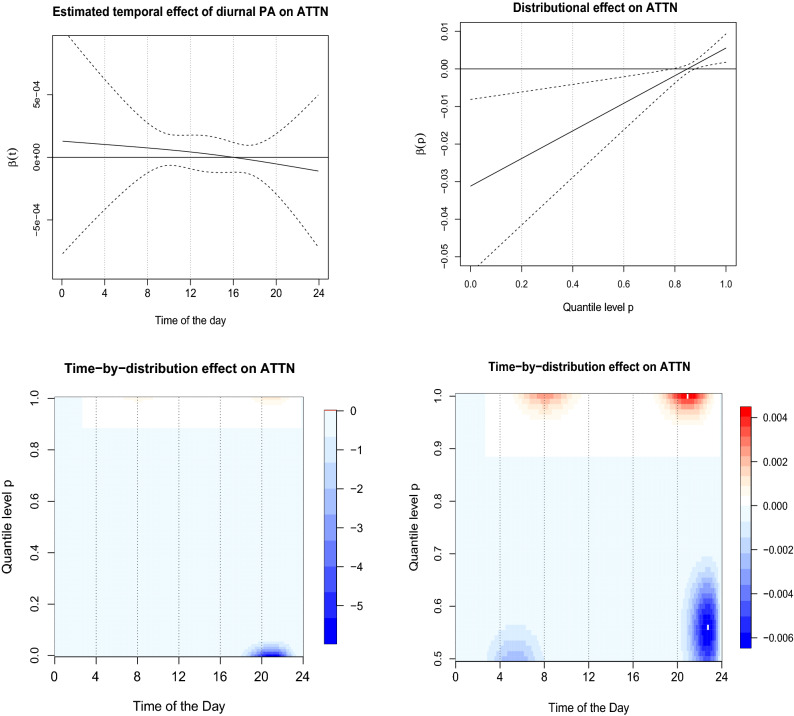
The estimated effects of the different PA metrics (Model 2-4) on ATTN score. Estimated temporal effect (solid line) $\beta (t)$ (top left). Estimated distributional effect $\beta (p)$ (top right). Estimated bivariate effect $\beta (t,p)$ of time-by-distribution PA surface (bottom left). The same plot (zoomed-in) with *p* restricted to the distributional domain (0.5, 1) (bottom right).

Table 3 presents the result of the analyses from these four modelling approaches. The association between average PA and attention is not found to be significant at $\alpha =0.05$ level. Adjusted R-squared of the model is reported to be 0.161 and is set as the benchmark for comparison with the other approaches. Although the diurnal curves of PA were not found to be significant ($\alpha =0.05$ level), the estimated quantile-function effect is significant. The estimated regression coefficient $\beta (p)$ is shown in Fig. 6 (top right panel). It shows that $\beta (p)$ creates a contrast between a higher quantile levels ($p > 0.8$) and lower quantile levels ($p < 0.8$). Specifically, an increase in higher quantile of PA is found to be associated with higher performance on attention. Although one needs to be cautious in interpreting the results as subject- specific quantiles of PA are mostly zero below the quantile level $p<0.5$ as illustrated in Fig. 1. A $35\%$ increase in the adjusted R-squared is observed using DDA with subject-specific quantile functions of PA compared to the benchmark model.

**Table 3:** The results of modelling attention score and physical activity using Model 1–4 with an adjustment for age, sex, and education.

	*Dependent variable : ATTN score*			
	Model 1	Model 2	Model 3	Model 4
Intercept	− 1.423	− 1.157	− 2.045**	− 3.696***
	(0.929)	(0.960)	(0.927)	(0.988)
Age	0.002	− 0.001	0.006	0.021*
	(0.011)	(0.011)	(0.010)	(0.011)
Sex	− 0.354**	− 0.349**	− 0.443***	− 0.476***
	(0.150)	(0.150)	(0.150)	(0.134)
Education	0.083***	0.080***	0.072***	0.069***
	(0.023)	(0.023)	(0.023)	(0.021)
${\bar{X}}_{i}$	0.0005	NA	NA	NA
	(0.0005)			
$X_i(t)$	NA	${\hat{\beta }}(t)$	NA	NA
$$Q_i(p)$$	NA	NA	$${\hat{\beta }}(p)^{**}$$	NA
$$Q_{i}(t,p)$$	NA	NA	NA	$${\hat{\beta }}(t,p)^{***}$$
Observations	92	92	92	92
Adjusted $\hbox {R}^{2}$	0.161	0.163	0.218	0.378
cv $\hbox {R}^{2}$	0.167	0.189	0.240	0.333

The standard deviation of the estimated coefficients for the scalar predictors are indicated in the parenthesis. Predictors: model 1-scalar average PA, model 2-diurnal PA curves, model 3-quantile functions, model 4-SOTDR with time-by-distribution data objects. All models are adjusted for age, sex, years of education.

*$${p}<0.1$$; **$${p}<0.05$$; ***$${p}<0.01$$.

The estimated bivariate coefficient $\beta (t,p)$, capturing the TD effect on attention is displayed in Fig. 6 (bottom left panel). Increased maximal capacity of PA during the morning ($\sim$ 7 a.m.–10 a.m.) and in the evening ($\sim$ 8 p.m.–10 p.m.) is found to be associated with higher attention score after adjusting for age, sex and years of education. Importantly, when quantile levels are constrained to be above 0.5 (re-estimated $\beta (t,p)$ is shown in the bottom right), there are two contrasts between upper quantile levels ($p > 0.9$) and lower quantile levels ($0.5< p < 0.7$) which are not time-aligned and actually capture quantile contrast between adjacent time periods. The morning TD effect can be interpreted as lower level quantile activity centered around 4–6 a.m. are negatively associated and higher level quantile activity centered around 7–9 a.m. are positively associated with attention.

The evening TD effect can be interpreted higher level quantile activity centered around 8–10 p.m. are positively associated and lower level quantile activity centered around 10 p.m.–12 a.m. are negatively associated with with attention. Adjusted R-squared of SOTDR model using the time-by-distribution PA surface is reported to be 0.378, giving a $135\%$ gain from the benchmark model using average physical activity, demonstrating very strong time-by-distribution effect, compared to the non-significant average and diurnal effect and significant distributional effect. In terms of cross-validated R-squared also, we see a $99\%$ increase using the SOTDR approach compared to the benchmark model.

The results from the similar SOTDR analysis of verbal memory (VM) and executive function (EF) are presented in the Supplementary Tables 1, 2 and Supplementary Figures 1, 2 of the Supplementary Material. For both outcomes, we observed significant improvements in adjusted R-squared and CV R-squared.

### SOTDR-based scalar biomarkers

**Figure 7:**
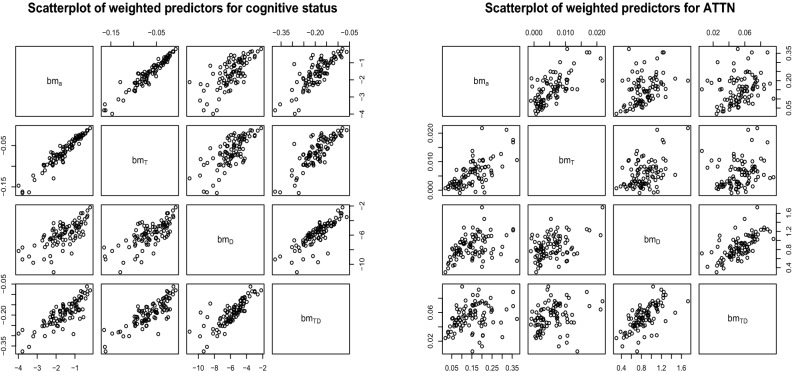
Scatterplots of the estimated weighted scores corresponding to the predictors average PA, diurnal PA curve, PA quantile function and time-by-distribution PA metric respectively for cognitive status (left) and ATTN (right). *Note*: $bm_a$ corresponds to average daily total count, $bm_T$ corresponds to temporal curves of PA, $bm_D$ corresponds to distribution representation (via quantile functions) of PA and $bm_{TD}$ corresponds to time-by-distribution representation of PA.

Estimates from SOTDR can be used to create simpler to use and interpretable scalar biomarkers. For example, based on the previously fitted models for an outcome of interest, one can calculate SOTDR biomarkers defined as $bm_{TD,i} =\int _{0}^{1} \int _{T}Q_i(t,p){\hat{\beta }}(t,p)dtdp$ and compare them with the biomarkers based on the average PA, diurnal curves of PA, and quantile functions of PA: $bm_{a,i} = {\bar{X}}_i{\hat{\beta }}$, $bm_{T,i} =\int _{T} X_i(t){\hat{\beta }}(t)dt$, $bm_{D,i} = \int _{0}^{1} Q_i(p){\hat{\beta }}(p)dp$. Figure 7 displays the scatterplot matrix for all four types of biomarkers to discriminate either cognitive status (left) or attention score (right). Although, they are mostly positively correlated, the large amount of spread indicates that they likely capture somewhat different aspects of PA.

## Discussion

In this paper, we have proposed to use subject-specific time-by-distribution data objects to capture and model temporally local distributional information in wearable data. We then developed a scalar on time-by-distribution regression that handles TD objects as predictors. We have also provided an alternative and parsimonious representation of the time-by-distribution objects in terms of time-varying L-moments, robust rank-based analogs of traditional moments. This representation allowed us to illustrate that SOTDR generalizes SOFR.

Our approach revealed novel insights into the associations between distributional and diurnal aspects of physical activity and various domains of cognitive function and Alzheimer’s disease status. The time-by-distribution representation provided better discrimination between the CNC and AD participants. Our results revealed strong associations between temporally local distributional aspects of PA across the day and clinical cognitive scales impacted in early AD, especially, attention. These results highlight the potential value of designing and testing physical activity interventions targeting a specific time of the day, in the early stages of AD. As there may be times of the day when cognitively impaired individuals are most alert^59,60^, it might be specifically suited for individual specific PA interventions. Note that, although, we have not established a causal direction here, it could also be that people with AD have poorer sleep, so are less active in the morning compared to cognitively normal controls. The maximal capacity of physical activity represents the reserve of an individual and our study has revealed strong and significant associations between cognitive performance and maximal PA levels, indicating changes in the reserve of a person might be sensitive to specific disease pathology and cognitive decline.

In this paper, we have proposed a two stage estimation approach of 1) using a LASSO penalty to identify the components of the stacked vectors $\mathbf {W}$ that are associated with the outcome 2) re-estimating the GLM model using components selected in Step 1. Step 2 does depend on components selected in Step 1, and our approach does not account for variability involved in selecting the components. This is not a limitation of the SOTDR model but of the current estimation approach that needs to address the smaller size of the application dataset. Note that for a larger dataset, this regularization in the estimation step will not be necessary. Also, methods for doing post-selection inference for LASSO (Lee et al. 2016; Taylor and Tibshirani 2018) may be extended to our framework in future work. A related concern is the penalization bias of LASSO which is known to shrink smaller coefficients to zero. An alternative would be to use adaptive LASSO^61^ or non-convex penalties such as SCAD^62^ or MCP^63^ which are known to overcome the penalization bias by adaptively relaxing the rate of penalization when the magnitude of the coefficient gets larger.

This paper opens interesting research questions on how to efficiently capture information with TD data objects. In our approach, we encoded distributional information via quantile functions, the use of other distributional representation such as CDF or hazard function could be explored in future work. In our application, the window length *h* for calculating $Q_i(t,p)$ and $L_i(t)$ was chosen to be consistent with the window size for diurnal curves. However, in other applications, an adaptive procedure of the choice of optimal window size *h* may be developed. Time registration or time-warping is often a desirable pre-processing step to make sure the amplitude and phase variations in functional data are properly separated^64–66^. This is especially important for wearable data which is often driven by subject-specific schedules and time preferences. Thus, pre-registration of TD objects is another exciting area of future research. We have focused on a linear effect of the TD data objects in this paper due to its simplicity, interpretability and connection with summary level modelling approaches. Accounting for the circular nature of the data may be another interesting direction. Future applications might benefit from considering nonlinear effects of the TD objects and this could be done via nonlinear extensions scalar-on-function regression models^17^. Another interesting area of research would be to extend and apply the proposed method for modelling longitudinal or multilevel data that at each visit generate distribution. To address day-to-day specific variation and account for weekly social structures, a possible approach could be to extend multilevel methods^16,67^ to TD objects or to employ a three dimensional day-by-time-by-distribution object $Q_i(d,t,p)$, with $d = Mon,Tue, Wed, Thu, Fri, Sat, Sun$. This approach, of course, would require more wearable data at subject level. Shared parameter model^68^ can also be useful for accommodating possible systematic differences across days of the week or times of the wday due to exogenous factors. The bivariate time-by-distribution object in the SOTDR framework could be modelled using a semi-parametric model and then linked to the scalar outcome via one or more shared latent parameters. These modifications that can be done in future work could help us to better understand associations between human health and temporal and distributional aspects of daily physical activity.

## Supplementary Information

Supplementary Information.

## Data Availability

Illustration of the proposed framework via R^51^, along with the dataset analyzed, is available online with this article and on Github at https://github.com/rahulfrodo/SOTDR.
