# Supplementary material for: Scalar on time-by-distribution regression and its application for modelling associations between daily-living physical activity and cognitive functions in Alzheimer’s Disease
Source: Sci Rep. 2022 Jul 7;12:11558. doi: 10.1038/s41598-022-15528-5 (PMC9263176; doi:10.1038/s41598-022-15528-5)
Supplement: Supplementary file 1 — Supplementary Information. [file 41598_2022_15528_MOESM1_ESM.pdf]

Supplementary Material for Scalar on time-by-distribution regression and its application for modelling associations between daily-living physical activity and cognitive functions in Alzheimer's Disease

Rahul Ghosal[1,*], Vijay R. Varma[2], Dmitri Volfson[3], Jacek Urbanek[4], Jeffrey M. Hausdorff[5,7,8], Amber Watts[6], and Vadim Zipunnikov[1]

[1]Department of Biostatistics, Johns Hopkins Bloomberg School of Public Health, Baltimore, Maryland USA
[2]National Institute on Aging (NIA), National Institutes of Health (NIH), Baltimore, Maryland, USA
[3]Neuroscience Analytics, Computational Biology, Takeda, Cambridge, MA, USA
[4]Department of Medicine, Johns Hopkins University School of Medicine, Baltimore Maryland, USA
[5]Center for the Study of Movement, Cognition and Mobility, Neurological Institute, Tel Aviv Sourasky Medical Center, Tel Aviv, Israel
[6]Department of Psychology, University of Kansas, Lawrence, KS, USA
[7]Department of Physical Therapy, Sackler Faculty of Medicine, and Sagol School of Neuroscience, Tel Aviv University, Tel Aviv, Israel
[8]Rush Alzheimer's Disease Center and Department of Orthopedic Surgery, Rush University Medical Center, Chicago, USA
[*]rahulghosal3@gmail.com

ABSTRACT

Contains Supplementary Tables and Figures referenced in the main paper.

1 Supplementary Tables

Table 1. Results from modelling cognitive score of VM on age, sex, education and physical activity metrics using Model 1-4. The standard deviation of the estimated coefficients for the scalar predictors are indicated in the parenthesis. Model 1: summary level modelling using average PA, Model 2: Temporal modelling using diurnal PA, Model 3: Distributional modelling using PA quantile function, Model 4: Joint modelling using PA time-by-distribution bivariate surface.

	Dependent variable : VM score			
	Model 1	Model 2	Model 3	Model 4
Intercept	-2.329	-1.561	-3.635^{*}	-3.786*
	(1.950)	(2.006)	(1.948)	(2.061)
age	-0.009	-0.017	0.001	-0.0001
	(0.023)	(0.023)	(0.022)	(0.023)
Sex	-1.355^{***}	-1.338^{***}	-1.533^{***}	-1.35^{***}
	(0.315)	(0.313)	(0.312)	(0.306)
education	0.164^{***}	0.156^{***}	0.142^{***}	0.126^{***}
	(0.049)	(0.049)	(0.048)	(0.047)
$\bar{X}_i$	0.003^{***}	NA	NA	NA
	(0.001)			
$X_i(t)$	NA	$\hat{\beta}(t)^{***}$	NA	NA
$Q_i(p)$	NA	NA	$\hat{\beta}(p)^{***}$	NA
$Q_i(t,p)$	NA	NA	NA	$\hat{\beta}(t,p)^{***}$
Observations	92	92	92	92
Adjusted R^2	0.331	0.338	0.375	0.413
cv R^2	0.334	0.369	0.405	0.388
Note:	$^{*}p<0.1$; $^{**}p<0.05$; $^{***}p<0.01$			

Table 2. Results from modelling cognitive score of EF on age, sex, education and physical activity metrics using Model 1-4. The standard deviation of the estimated coefficients for the scalar predictors are indicated in the parenthesis. Model 1: summary level modelling using average PA, Model 2: Temporal modelling using diurnal PA, Model 3: Distributional modelling using PA quantile function, Model 4: Joint modelling using PA time-by-distribution bivariate surface.

	Dependent variable : EF score			
	Model 1	Model 2	Model 3	Model 4
Intercept	-2.479	-1.944	-3.070^{**}	-3.760^{**}
	(1.492)	(1.539)	(1.531)	(1.612)
age	-0.0002	-0.006	0.004	0.009
	(0.017)	(0.018)	(0.017)	(0.018)
Sex	-1.063^{***}	-1.051^{***}	-1.141^{***}	-1.094^{***}
	(0.241)	(0.240)	(0.245)	(0.230)
education	0.141^{***}	0.136^{***}	0.132^{***}	0.116^{***}
	(0.037)	(0.037)	(0.038)	(0.036)
$\bar{X}_i$	0.002^{***}	NA	NA	NA
	(0.001)			
$X_i(t)$	NA	$\hat{\beta}(t)^{***}$	NA	NA
$Q_i(p)$	NA	NA	$\hat{\beta}(p)^{***}$	NA
$Q_i(t,p)$	NA	NA	NA	$\hat{\beta}(t,p)^{***}$
Observations	92	92	92	92
Adjusted R^2	0.337	0.341	0.347	0.411
cv R^2	0.351	0.361	0.371	0.378
Note:	*p<0.1; **p<0.05; ***p<0.01			

2 Supplementary Figures

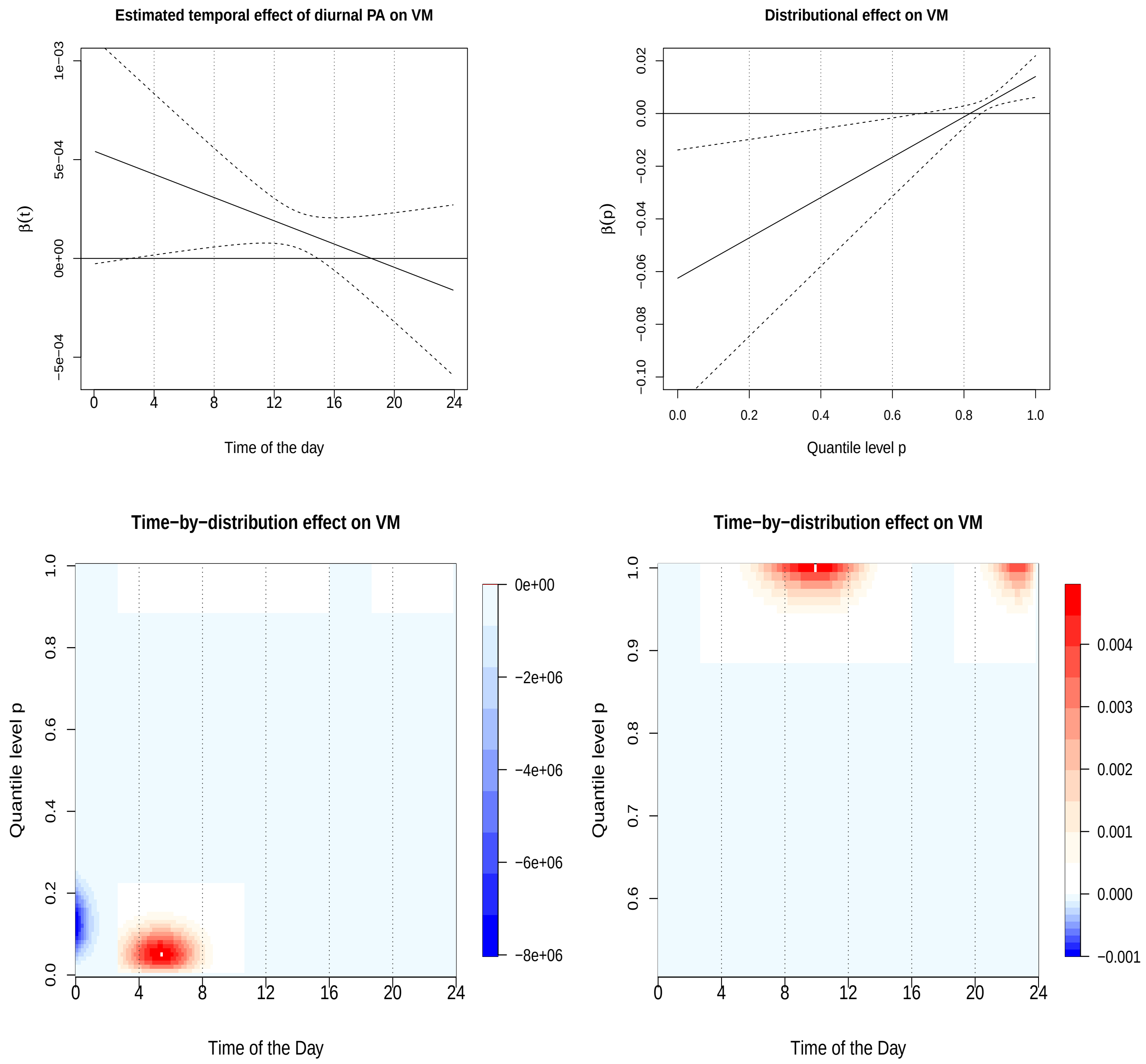

Figure 1. The estimated effects of the different PA metrics (Model 2-4) on VM score. Estimated temporal effect (solid line) $\beta(t)$ (top left). Estimated distributional effect $\beta(p)$ (top right). Estimated bivariate effect $\beta(t,p)$ of time-by-distribution PA surface (bottomleft). The same plot with p restricted to $(0.5,1)$ (bottomright). Higher maximal PA during the morning and night are found be associated with a higher score of VM. Higher minimal PA during early morning also appears to be associated with higher score of VM, caution should be taken when interpreting the results for $p < 0.5$.

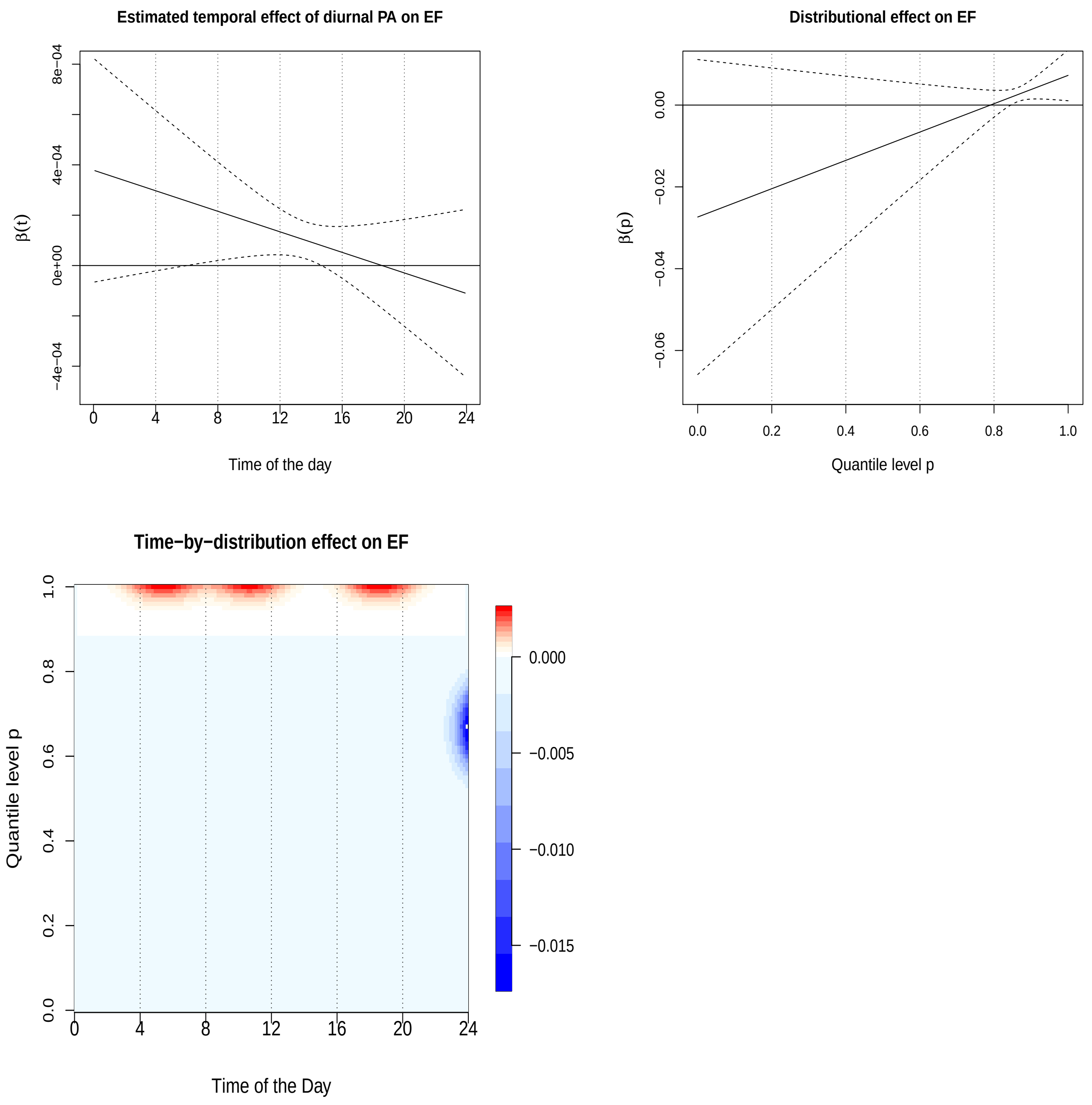

Figure 2. The estimated effects of the different PA metrics (Model 2-4) on EF score. Estimated temporal effect (solid line) $\beta(t)$ (top left). Estimated distributional effect $\beta(p)$ (top right). Estimated bivariate effect $\beta(t,p)$ of time-by-distribution PA surface (bottomleft). Higher maximal PA during the morning and evening is found to be associated with higher EF score.
